# Crystal structures of ethyl 6-(4-methyl­phen­yl)-4-oxo-4*H*-chromene-2-carboxyl­ate and ethyl 6-(4-fluoro­phen­yl)-4-oxo-4*H*-chromene-2-carboxyl­ate

**DOI:** 10.1107/S2056989015022781

**Published:** 2016-01-01

**Authors:** Ligia R. Gomes, John Nicolson Low, Carlos Fernandes, Alexandra Gaspar, Fernanda Borges

**Affiliations:** a FP-ENAS-Faculdade de Ciências de Saúde, Escola Superior de Saúde da UFP, Universidade Fernando Pessoa, Rua Carlos da Maia, 296, P-4200-150 Porto, Portugal; b REQUIMTE, Departamento de Química e Bioquímica, Faculdade de Ciências da Universidade do Porto, Rua do Campo Alegre, 687, P-4169-007, Porto, Portugal; c Department of Chemistry, University of Aberdeen, Meston Walk, Old Aberdeen, AB24 3UE, Scotland; d CIQ/Departamento de Química e Bioquímica, Faculdade de Ciências, Universidade do Porto, 4169-007 Porto, Portugal

**Keywords:** crystal structure, drug design, chromone, conformation supra­molecular structure

## Abstract

The crystal structures of two chromone derivatives, *viz.* ethyl 6-(4-methyl­phen­yl)-4-oxo-4*H*-chromene-2-carboxyl­ate, C_19_H_16_O_4_, (**1**), and ethyl 6-(4-fluoro­phen­yl)-4-oxo-4*H*-chromene-2-carboxyl­ate C_18_H_13_FO_4_, (**2**), have been determined: (**1**) crystallizes with two mol­ecules in the asymmetric unit. A comparison of the dihedral angles beween the mean planes of the central chromone core with those of the substituents, an ethyl ester moiety at the 2-position and a *para*-substituted phenyl ring at the 6-position shows that each mol­ecule differs significantly from the others, even the two independent mol­ecules (*a* and *b*) of (**1**). In all three mol­ecules, the carbonyl groups of the chromone and the carboxyl­ate are *trans*-related. The supra­molecular structure of (**1**) involves only weak C—H⋯π inter­actions between H atoms of the substituent phenyl group and the phenyl group, which link mol­ecules into a chain of alternating mol­ecules *a* and *b*, and weak π–π stacking inter­actions between the chromone units. The packing in (**2**) involves C—H⋯O inter­actions, which form a network of two inter­secting ladders involving the carbonyl atom of the carboxyl­ate group as the acceptor for H atoms at the 7-position of the chromone ring and from an *ortho*-H atom of the exocyclic benzene ring. The carbonyl atom of the chromone acts as an acceptor from a *meta*-H atom of the exocyclic benzene ring. π–π inter­actions stack the mol­ecules by unit translation along the *a* axis.

## Abstract 2

The crystal structures of two chromone derivatives are described, one of which has two independent mol­ecules in the asymmetric unit. A comparison of the dihedral angles between the mean planes of the central chromone core with those of the substituents shows that each mol­ecule differs significantly from the others.

## Chemical context   

Benzo­pyran derivatives represent a large class of natural and synthetic heterocycles that are often linked to a broad array of biological activities, (Gaspar *et al.*, 2014 ▸, 2015 ▸). Within this vast class of compounds, the chromone core has emerged as a privileged structure for drug discovery and development programs (Welsch *et al.*, 2010 ▸). Chemically, the chromone scaffold is a rigid benzoannelated γ-pyrone ring, which can be modulated by diversity-oriented synthesis, (Gaspar *et al.*, 2015 ▸; Welsch *et al.*, 2010 ▸; Ko *et al.*, 2006 ▸; Nicolaou *et al.*, 2000 ▸), exhibiting a diversity of pharmacological properties such as anti-inflammatory, anti­microbial and anti­cancer among others (Gaspar *et al.*, 2015 ▸). The application of chromones as a valid scaffold for the development of therapeutic solutions for aging-related diseases is still an emerging field, even though the data acquired indicate their importance in the development of new drug candidates for targets ascribed with respect to Alzheimer’s and Parkinson’s diseases, namely as adenosine receptors ligands (Cagide *et al.*, 2015*a* ▸) and/or as mono­amino oxidase B inhibitors, (Cagide *et al.*, 2015*b* ▸).
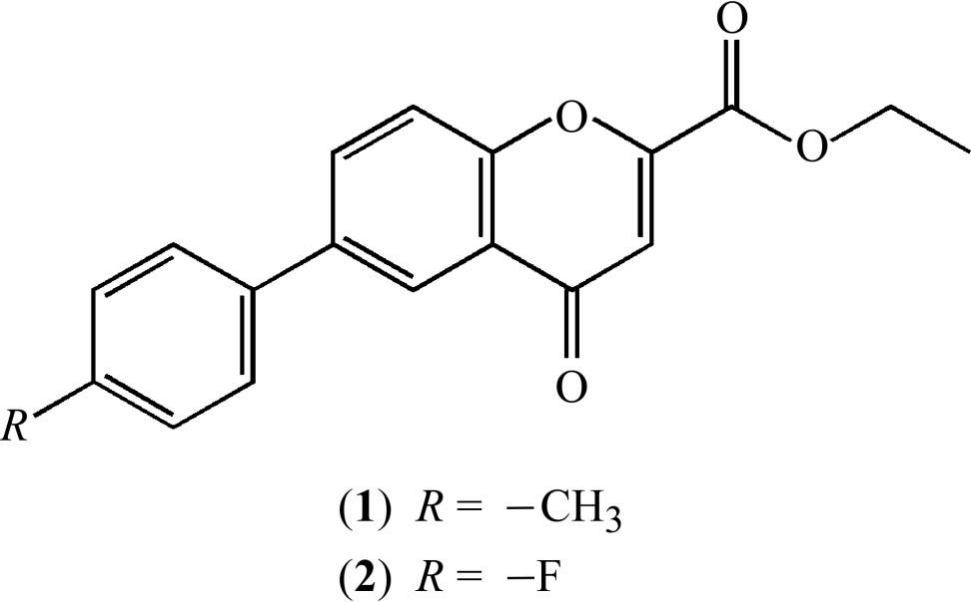


Within this framework, our project has been focused on the discovery of new chemical entities based on a chromone scaffold. Herein we describe the crystal structures of two new chromone derivatives, *viz*. ethyl-6-(4-methyl­phen­yl)-4-oxo-4*H*-chromene-2-carboxyl­ate (**1**) and ethyl-6-(4-fluoro­phen­yl)-4-oxo-4*H*-chromene-2-carboxyl­ate (**2**).

## Mol­ecular Geometry   

**Figure 1:**
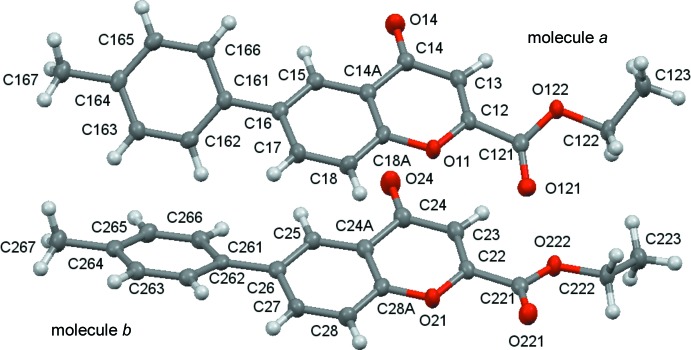
A view of the asymmetric unit of (**1**), with displacement ellipsoids drawn at the 70% probability level.

Ellipsoid plots of the mol­ecules are given in Figs. 1 ▸ and 2 ▸. Compound (**1**) crystallizes with two mol­ecules (*a* and *b*) in the asymmetric unit.

The mol­ecules consist of a central chromone core with an ethyl­ester substituent at the 2-position and a *p*-substituted phenyl group at the 6-position of the chromone ring system. Those constitutive fragments are essentially planar, therefore the major contribution to the definition of the mol­ecular conformations are the rotations around the C—C bonds that connect the substituents to the chromone ring. As such, the analysis of the mol­ecular geometry will be based on the values for the dihedral angles between the mean planes of the chromone and the phenyl ring (θ_Chr–Phe_) and the chromone and the ethyl carboxyl­ate moiety (θ_Chr–carboxlylate_), Table 1 ▸. As can be seen, the dihedral angles for mol­ecules *a* and *b* of (**1**) are significantly different from each other. An overlay fit using the quaternion transformation method (Mackay, 1984 ▸) shows that mol­ecule i inverts on mol­ecule ii where the weighted/unit weight r.m.s. fits are 0.090/0.089 Å for 23 atoms. The largest individual displacement is 0.169 Å (O14/O24 pair). The r.m.s. bond fit is 0.0021 Å and the r.m.s. angle fit is 0.376°. These values show that, in spite of the large differences in the dihedral angles, the mol­ecules are quite similar overall.

**Figure 3:**
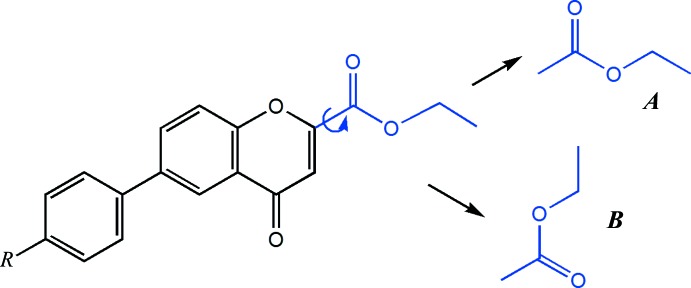
The relative position of the ethyl carboxyl­ate residue with respect to the chromone ring. Mol­ecules may have any conformation between two possible extremes: conformation *A* where the carbonyl groups are *trans*-related and conformation *B* where they are *cis*-related.

Considering the relative position of the ethyl carboxyl­ate residue with respect to the chromone ring as may be seen in Fig. 3 ▸, the mol­ecules may have any conformation between two possible extremes: conformation *A* where the carbonyl groups are *trans*-related and conformation *B* where they are *cis*-related. A theoretical calculation made with *Gaussian03* (Frisch *et al.*, 2004 ▸) at the B3LYP /631++(d,p) level shows that the energy associated with each of the boundary conformations is similar in adiabatic conditions [see supporting information; the B3LYP model combines the hybrid exchange functional of Becke (1997 ▸) with the gradient-correlation functional of Lee *et al.* (1988 ▸) and the split-valence polarized 6-311+G(d, p) basis set (Hehre *et al.*, 1986 ▸)]. Thus the adopted conformation in the solid state, with a geometry closer to *A* where the degree of twist lies between 9 and 21° (as measured by dihedral angles) may be due to packing factors. Preliminary results for the structures of similar compounds such as 6-(phen­yl)-4-oxo-4*H*-chromene-2-carboxyl­ate, 6-(4-meth­oxy­phen­yl)-4-oxo-4*H*-chromene-2-carboxyl­ate and 6-(4-3,4-di­meth­oxy­phen­yl)-4-oxo-4*H*-chromene-2-carboxyl­ate indicate that the major components have the same *trans* conformation as described above. These structures are imprecisely determined (the crystal quality was poor and the structures appeared to be intra­ctably disordered).

The rotation around the C(phen­yl)—C(chromone) bond is higher than the rotation around the C(chromone)—C(carb­oxy­eth­yl) bond for all of the three mol­ecules. This rotation may also contribute to the mol­ecular packing since, in the absence of electronically crowded substituents in the *o*- or *m*- positions, the phenyl substituent does not impose steric hindrance with respect to the chromone ring.

## Supra­molecular structures   

In the absence of strong hydrogen-bond donors, the supra­molecular structures depend on weak C—H⋯O hydrogen bonds and C—H⋯π and very weak π–π inter­actions.

**Figure 4:**
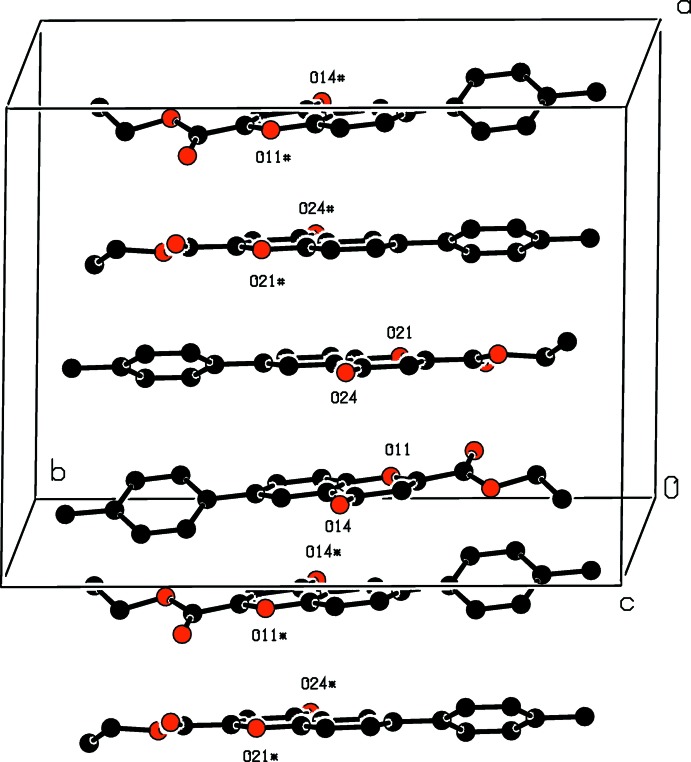
A view showing the stacking of the mol­ecules along the *a* axis. Symmetry codes: (*) −*x*, −*y* + 1, −*z* + 1; (#) −*x* + 1, −*y* + 1, −*z* + 1. H atoms are omitted.

In (**1**) there are no weak C—H⋯O inter­actions and aromatic inter­actions appear to play the major role in the establishment of the packing. There are two T-shaped C—H⋯π inter­actions, one between C162 and the centroid of the phenyl ring with pivot atom C261, *Cg*(C261) within the selected asymmetric unit, and the other between C262 and the centroid of the phenyl ring with pivot atom C161, *Cg*(C161)(*x*, 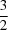 − *y*, −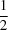 + *z*), Table 2 ▸. This forms a chain of alternating glide-related asymmetric units which runs parallel to the *c* axis. Within the asymmetric unit, the shortest packing contact is between the rings containing C15 and C25 and has a value of 4.2901 (9) Å, with an average perpendicular distance between the planes of 3.5350 Å and an angle between the planes of 6.46 (7)°, suggesting a possible very weak π–π inter­action. Centrosymmetrically related pairs of mol­ecule i form π–π stacked pairs, as do centrosymmetric pairs of mol­ecule ii, Table 3 ▸. These base-paired units form a column of mol­ecule along the *a* axis, Fig. 4 ▸.

**Figure 5:**
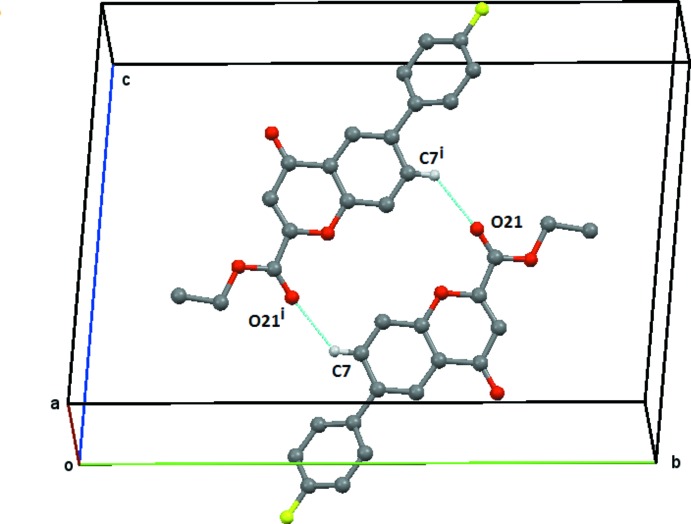
Compound (**2**), view of the C7—H7⋯O21 centrosymetric 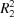
(16) ring structure centred on (0, ½, ½). Atoms labelelled with a postscript,(i), are in mol­ecules at (−*x*, −*y* + 1, −*z* + 1). Hydrogen atoms not involved in the hydrogen bonding are omitted.

**Figure 6:**
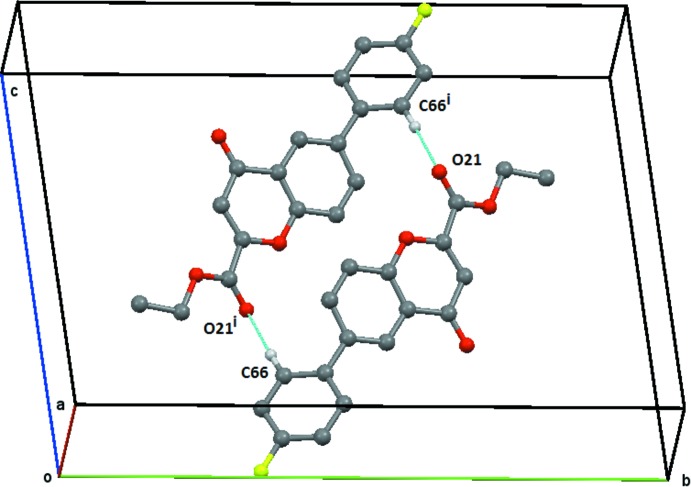
Compound (**2**), view of the C66—H66⋯O21 centrosymetric 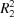
(22) ring structure centred on (½, ½, ½). Symmetry code: (i) = −*x* + 1, −*y* + 1, −*z* + 1. H atoms not involved in the hydrogen bonding are omitted.

**Figure 7:**
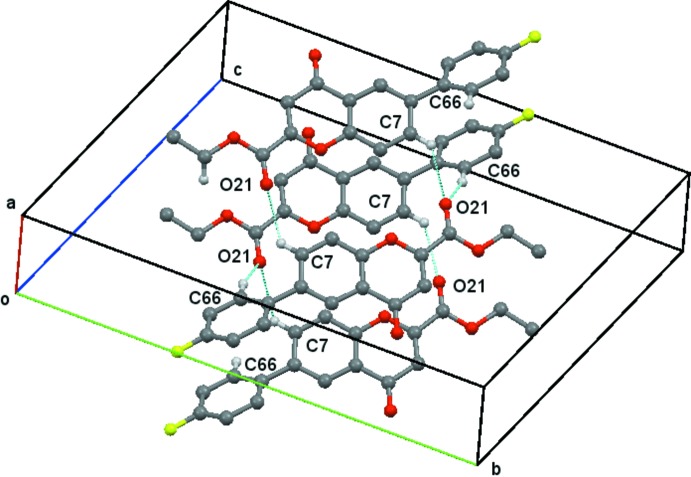
Compound (**2**), the combined ring structure formed by the combination of the ring structures in Figs. 4 ▸ and 5 ▸. This chain of rings extends along the *a* axis. H atoms not involved in the hydrogen bonding are omitted.

**Figure 8:**
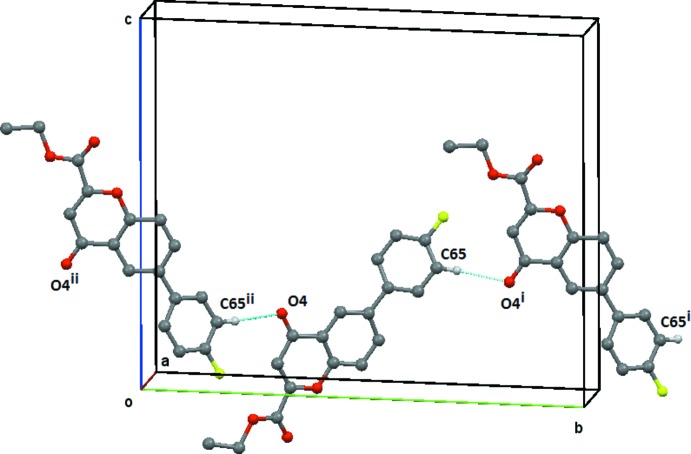
Compound (**2**), the simple *C*9 chain formed by the C65—H65⋯O4 weak hydrogen bond. This chain of rings extends along the *a* axis and is generated by the twofold screw axis at (1, *y*, 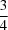). Symmetry codes: (i) −*x* + 2, *y* + 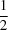, −*z* + 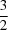; (ii) −*x* + 2, *y* − 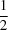, −*z* + 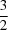. H atoms not involved in the hydrogen bonding are omitted.

**Figure 9:**
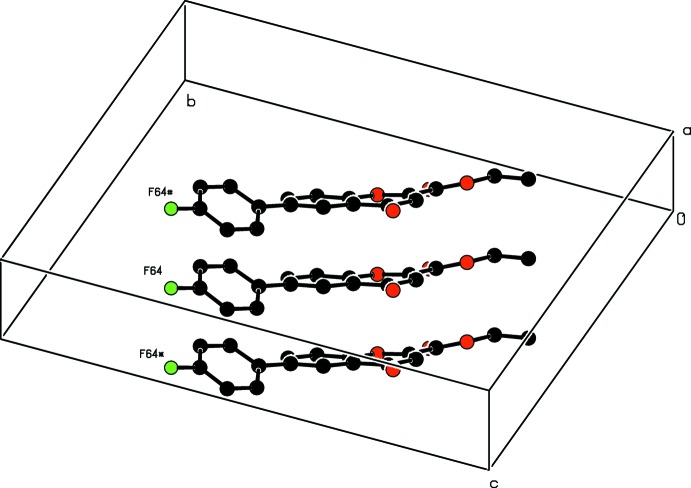
A view showing the stacking of the mol­ecules along the *a* axis. Symmetry codes: (*) *x* − 1, *y*, *z*; (#) *x* + 1, *y*, *z* + 1. H atoms are omitted.

In contrast, compound (**2**) has a more intricate supra­molecular structure, based on C—H⋯O and π–π inter­actions (Tables 3 ▸ and 4 ▸). Both carb­oxy­lic oxygen atoms (O21 and O4) act as acceptors of C—H⋯O hydrogen bonds. Atom O21 is involved in two centrosymmetrically linked ring structures. In one of these, the C7—H7⋯O21(−*x*, −*y* + 1, −*z* + 1) hydrogen bond forms an 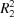
(16) ring, Fig. 5 ▸, and in the other the C66—H66⋯O21(−*x* + 1, −*y* + 1, −*z* + 1) hydrogen bond forms an 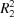
(22) ring (Fig. 6 ▸). These inter­actions combine to link the mol­ecules into zigzag chains of rings which run parallel to the *a* axis, Fig. 7 ▸. These are linked to form a three-dimensional network by the C65—H65⋯O4(−*x* + 2, *y* + 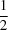, −z + 3/2) weak hydrogen bond formed by the action of the twofold screw axis at (1, *y*, 3/4), Fig. 8 ▸. The mol­ecules are π–π stacked above each other with unit translation along the *a* axis, Table 3 ▸ and Fig. 9 ▸.

The inter­molecular inter­actions probably account for the significant difference (about 36 K) in the melting points for these compounds [411–418 K for (**1**) and 446–455 K for (**2**)]. They also may have an influence in the conformations of the mol­ecules since in (**2**) the atoms in the carb­oxy­ethyl group are involved either as donors or acceptors; these inter­actions may constrain the conformation of the orientation of the carb­oxy­ethyl moiety.

## Synthesis and crystallization   

Compounds (**1**) and (**2**) were obtained, in moderate yields, by a two-step synthetic procedure. In the first step, the required phenyl­aceto­phenone derivatives were obtained from 5′-bromo-2′-hy­droxy­aceto­phenone by a Suzuki C–C cross-coupling reaction assisted by microwave (MW) heating (Soares *et al.*, 2015 ▸). In the second step, the phenyl­aceto­phenone derivatives were converted in the corresponding chromones *via* an intra­molecular Claisen condensation reaction accomplished with diethyl oxalate in the presence of ethano­lic sodium ethoxide and cyclization under acidic conditions of the inter­mediate formed *in situ*.

Ethyl 6-(4-methyl­phen­yl)-4-oxo-4*H*-chromene-2-carboxyl­ate (**1**). Overall yield 50.7%; m.p. 411–418 K. Crystallization: ethyl acetate to form colourless prisms.

Ethyl 6-(4-fluoro­phen­yl)-4-oxo-4*H*-chromene-2-carboxyl­ate (**2**). Overall yield 55.9%; m.p. 446–455 K. Crystallization: ethyl acetate, to form colourless needles.

## Refinement   

Crystal data, data collection and structure refinement details are summarized in Table 5 ▸. H atoms were treated as riding atoms with C—H(aromatic) = 0.95 Å, with *U*_iso_ = 1.2*U*_eq_(C) and *C*—H(meth­yl) = 0.98 Å with *U*_iso_ = 1.5*U*_eq_(C).

## Supplementary Material

Crystal structure: contains datablock(s) general, 1, 2. DOI: 10.1107/S2056989015022781/hb7543sup1.cif

Structure factors: contains datablock(s) 1. DOI: 10.1107/S2056989015022781/hb75431sup2.hkl

Structure factors: contains datablock(s) 2. DOI: 10.1107/S2056989015022781/hb75432sup3.hkl

Supporting information file. DOI: 10.1107/S2056989015022781/hb75431sup4.cml

Supporting information file. DOI: 10.1107/S2056989015022781/hb75432sup5.cml

Supporting information file. DOI: 10.1107/S2056989015022781/hb7543sup6.pdf

CCDC references: 1439394, 1439393

Additional supporting information:  crystallographic information; 3D view; checkCIF report

## Figures and Tables

**Figure 2:**
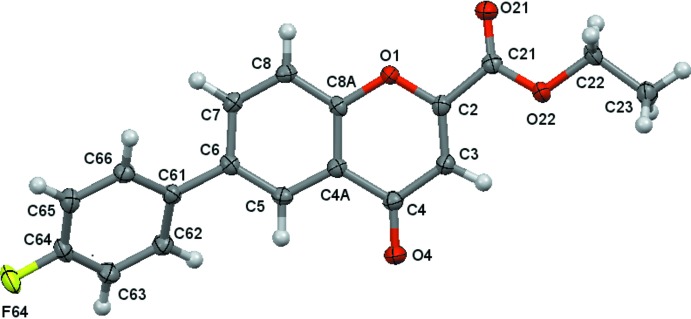
A view of the asymmetric unit of (**2**), with displacement ellipsoids drawn at the 70% probability level.

**Table 1:** Selected dihedral angles (°) θ**_Chr–C3ring_** is the dihedral angle between the mean planes of the chromene and the phenyl ring. θ**_Chr–C6ester_** is the dihedral angle between the mean planes of the chromone ring and the plane defined by the ester atoms attached to C2 but not including it. θ**_Chr–OCO_** is the dihedral angle between the mean planes of the chromone ring and the OCO atoms of the ester.

Compound	θ_Chr–Phe_	θ_Chr–carboxyl­ate_	θ_Chr–OCO_
(**1**) molecule *a*	32.8754)	23.23 (7)	21.16 (16)
(**1**) molecule *b*	24.14 (5)	14.191 (7)	12.16 (17)
(**2**)	36.05 (5)	9.52 (6)	12.97 (13)

**Table 2:** Hydrogen-bond geometry (Å, °) for (**1**)

*D*—H⋯*A*	*D*—H	H⋯*A*	*D*⋯*A*	*D*—H⋯*A*
C162—H162⋯*Cg*(C261)	0.95	2.85	3.4914 (15)	126
C262—H262⋯*Cg*(C161)^i^	0.95	2.84	3.5408 (4)	131

Symmetry code: (i) 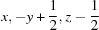.

**Table 3:** Selected π–π contacts and short inter­molecular contacts (Å, °) In compound (**1**), *Cg*1, *Cg*2, *Cg*5 and *Cg*6 are the centroids of the rings containing atoms O11, C15, O21 and C25, respectively. In compound (**2**), *Cg*1, *Cg*2 and *Cg*6 are the centroids of the rings containing atoms O1, C5 and C61. Values marked with an asterisk are average perpendicular distances and angles between the planes.

Compound	contacts	distance	perp. distance	slippage/angle*
(**1**)	*Cg*1⋯*Cg*2^i^	3.7338 (8)	3.503*	0.45*
	*Cg*2⋯*Cg*2^i^	3.7226 (8)	3.5040 (6)	1.257
	*Cg*5⋯*Cg*6^ii^	3.6743 (9)	3.824*	0.98*
	*Cg*6⋯*Cg*6^ii^	3.9299 (9)	3.5762 (6)	1.630
(**2**)	*Cg*1⋯*Cg*1^iii^	3.8521 (7)	3.3989 (4)	1.813
	*Cg*2⋯*Cg*2^iii^	3.8521 (7)	3.3957 (4)	1.819
	*Cg*3⋯*Cg*3^iii^’	3.8521 (7)	3.5811 (5)	1.419

Symmetry codes: (i) −*x*, 1 − *y*, 1 − *z*; (ii) 1 − *x*, 1 − *y*, 1 − *z*; (iii) 1 + *x*, *y*, *z*.

**Table 4:** Hydrogen-bond geometry (Å, °) for (**2**)

*D*—H⋯*A*	*D*—H	H⋯*A*	*D*⋯*A*	*D*—H⋯*A*
C7—H7⋯O21^i^	0.95	2.47	3.1977 (13)	133
C65—H65⋯O4^ii^	0.95	2.50	3.4447 (13)	175
C66—H66⋯O21^iii^	0.95	2.53	3.4425 (13)	162

Symmetry codes: (i) 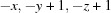; (ii) 
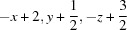
; (iii) 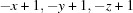.

**Table 5:** Experimental details

	(**1**)	(**2**)
Crystal data		
Chemical formula	C_19_H_16_O_4_	C_18_H_13_FO_4_
*M*_r_	308.32	312.28
Crystal system, space group	Monoclinic, *P*2_1_/*c*	Monoclinic, *P*2_1_/*c*
Temperature (K)	100	100
*a*, *b*, *c* (Å)	14.7129 (11), 18.9613 (13), 11.3031 (6)	3.8521 (2), 20.6970 (15), 17.5478 (11)
β (°)	111.632 (7)	91.546 (1)
*V* (Å^3^)	2931.2 (4)	1398.52 (15)
*Z*	8	4
Radiation type	Mo *K*α	Mo *K*α
μ (mm^−1^)	0.10	0.11
Crystal size (mm)	0.20 × 0.09 × 0.05	0.42 × 0.02 × 0.01

Data collection		
Diffractometer	Rigaku Saturn724+	Rigaku Saturn724+
Absorption correction	Multi-scan (*CrystalClear-SM Expert*; Rigaku, 20112)	Multi-scan *CrystalClear-SM Expert* (Rigaku, 20112)
*T*_min_, *T*_max_	0.981, 0.995	0.954, 0.999
No. of measured, independent and observed [*I* > 2σ(*I*)] reflections	22133, 6680, 5311	16479, 3177, 2725
*R*_int_	0.033	0.035
(sin θ/λ)_max_ (Å^−1^)	0.649	0.650

Refinement		
*R*[*F* ^2^ > 2σ(*F* ^2^)], *wR*(*F* ^2^), *S*	0.038, 0.104, 1.11	0.031, 0.087, 0.98
No. of reflections	6680	3176
No. of parameters	419	209
H-atom treatment	H-atom parameters constrained	H-atom parameters constrained
Δρ_max_, Δρ_min_ (e Å^−3^)	0.33, −0.23	0.31, −0.20

Computer programs: *CrystalClear-SM Expert* (Rigaku, 2012 ▸), *SHELXT* (Sheldrick, 2015*a* ▸), *SHELXL2014* (Sheldrick, 2015*b* ▸), *OSCAIL* (McArdle *et al.*, 2004 ▸), *ShelXle* (Hübschle *et al.*, 2011 ▸), *Mercury* (Macrae *et al.*, 2006 ▸) and *PLATON* (Spek, 2009 ▸).

## supplementary crystallographic information

### (1) Ethyl 6-(4-methylphenyl)-4-oxo-4H-chromene-2-carboxylate Crystal data

**Table:**

C_19_H_16_O_4_	*F*(000) = 1296
*M**_r_* = 308.32	*D*_x_ = 1.397 Mg m^−^^3^
Monoclinic, *P*2_1_/*c*	Mo *K*α radiation, λ = 0.71075 Å
*a* = 14.7129 (11) Å	Cell parameters from 18061 reflections
*b* = 18.9613 (13) Å	θ = 3.0–27.5°
*c* = 11.3031 (6) Å	µ = 0.10 mm^−^^1^
β = 111.632 (7)°	*T* = 100 K
*V* = 2931.2 (4) Å^3^	Prism, colourless
*Z* = 8	0.20 × 0.09 × 0.05 mm

### (1) Ethyl 6-(4-methylphenyl)-4-oxo-4H-chromene-2-carboxylate Data collection

**Table:**

Rigaku Saturn724+ (2x2 bin mode) diffractometer	6680 independent reflections
Radiation source: Rotating Anode	5311 reflections with *I* > 2σ(*I*)
Confocal monochromator	*R*_int_ = 0.033
Detector resolution: 28.5714 pixels mm^-1^	θ_max_ = 27.5°, θ_min_ = 3.0°
profile data from ω–scans	*h* = −17→19
Absorption correction: multi-scan (*CrystalClear-SM Expert*; Rigaku, 20112)	*k* = −17→24
*T*_min_ = 0.981, *T*_max_ = 0.995	*l* = −14→14
22133 measured reflections	

### (1) Ethyl 6-(4-methylphenyl)-4-oxo-4H-chromene-2-carboxylate Refinement

**Table:**

Refinement on *F*^2^	0 restraints
Least-squares matrix: full	Hydrogen site location: inferred from neighbouring sites
*R*[*F*^2^ > 2σ(*F*^2^)] = 0.038	H-atom parameters constrained
*wR*(*F*^2^) = 0.104	*w* = 1/[σ^2^(*F*_o_^2^) + (0.0504*P*)^2^ + 0.4579*P*] where *P* = (*F*_o_^2^ + 2*F*_c_^2^)/3
*S* = 1.11	(Δ/σ)_max_ = 0.001
6680 reflections	Δρ_max_ = 0.33 e Å^−^^3^
419 parameters	Δρ_min_ = −0.23 e Å^−^^3^

### (1) Ethyl 6-(4-methylphenyl)-4-oxo-4H-chromene-2-carboxylate Special details

**Table:**

Geometry. All e.s.d.'s (except the e.s.d. in the dihedral angle between two l.s. planes) are estimated using the full covariance matrix. The cell e.s.d.'s are taken into account individually in the estimation of e.s.d.'s in distances, angles and torsion angles; correlations between e.s.d.'s in cell parameters are only used when they are defined by crystal symmetry. An approximate (isotropic) treatment of cell e.s.d.'s is used for estimating e.s.d.'s involving l.s. planes.

### (1) Ethyl 6-(4-methylphenyl)-4-oxo-4H-chromene-2-carboxylate Fractional atomic coordinates and isotropic or equivalent isotropic displacement parameters (Å^2^)

**Table:**

	*x*	*y*	*z*	*U*_iso_*/*U*_eq_	
O11	0.13984 (7)	0.39931 (4)	0.51267 (8)	0.0167 (2)	
O14	0.14523 (8)	0.46234 (5)	0.86202 (8)	0.0239 (2)	
O121	0.18698 (7)	0.26721 (4)	0.47772 (8)	0.0186 (2)	
O122	0.13729 (7)	0.23141 (4)	0.63468 (8)	0.0162 (2)	
C12	0.14765 (9)	0.35255 (6)	0.60658 (11)	0.0153 (3)	
C13	0.14901 (9)	0.37041 (6)	0.72208 (11)	0.0161 (3)	
H13	0.1540	0.3344	0.7827	0.019*	
C14	0.14293 (10)	0.44401 (6)	0.75658 (11)	0.0166 (3)	
C14A	0.13289 (9)	0.49463 (6)	0.65301 (11)	0.0150 (2)	
C15	0.12422 (9)	0.56769 (6)	0.66738 (11)	0.0156 (3)	
H15	0.1247	0.5855	0.7462	0.019*	
C16	0.11494 (9)	0.61435 (6)	0.56912 (11)	0.0147 (2)	
C17	0.11434 (10)	0.58625 (6)	0.45309 (11)	0.0171 (3)	
H17	0.1084	0.6175	0.3849	0.021*	
C18	0.12206 (10)	0.51512 (7)	0.43612 (11)	0.0176 (3)	
H18	0.1209	0.4972	0.3571	0.021*	
C18A	0.13157 (9)	0.46978 (6)	0.53635 (11)	0.0150 (3)	
C121	0.15961 (9)	0.27911 (6)	0.56400 (11)	0.0152 (3)	
C122	0.16128 (10)	0.15886 (6)	0.61422 (11)	0.0173 (3)	
H12A	0.2328	0.1535	0.6384	0.021*	
H12B	0.1288	0.1457	0.5235	0.021*	
C123	0.12554 (11)	0.11295 (6)	0.69613 (12)	0.0222 (3)	
H12C	0.1445	0.0639	0.6900	0.033*	
H12D	0.0542	0.1162	0.6671	0.033*	
H12E	0.1546	0.1287	0.7848	0.033*	
C161	0.10558 (9)	0.69191 (6)	0.58084 (11)	0.0141 (2)	
C162	0.14423 (9)	0.73832 (6)	0.51543 (11)	0.0160 (3)	
H162	0.1776	0.7200	0.4645	0.019*	
C163	0.13461 (9)	0.81085 (6)	0.52375 (11)	0.0168 (3)	
H163	0.1612	0.8412	0.4779	0.020*	
C164	0.08671 (9)	0.83989 (6)	0.59792 (11)	0.0162 (3)	
C165	0.04831 (9)	0.79352 (6)	0.66303 (11)	0.0164 (3)	
H165	0.0150	0.8120	0.7140	0.020*	
C166	0.05741 (9)	0.72085 (6)	0.65533 (11)	0.0157 (3)	
H166	0.0306	0.6906	0.7011	0.019*	
C167	0.07977 (10)	0.91884 (6)	0.60848 (12)	0.0208 (3)	
H16A	0.0176	0.9310	0.6167	0.031*	
H16B	0.0831	0.9412	0.5320	0.031*	
H16C	0.1341	0.9356	0.6836	0.031*	
O21	0.36690 (7)	0.39338 (4)	0.38688 (8)	0.0173 (2)	
O24	0.39990 (8)	0.46146 (5)	0.74405 (8)	0.0256 (2)	
O221	0.34285 (7)	0.25860 (4)	0.32365 (8)	0.0197 (2)	
O222	0.39836 (7)	0.22764 (4)	0.53103 (8)	0.0170 (2)	
C22	0.37715 (9)	0.34766 (6)	0.48321 (11)	0.0151 (3)	
C23	0.38783 (9)	0.36726 (6)	0.60153 (11)	0.0166 (3)	
H23	0.3942	0.3321	0.6639	0.020*	
C24	0.38986 (10)	0.44161 (6)	0.63652 (11)	0.0170 (3)	
C24A	0.37849 (9)	0.49097 (6)	0.53085 (11)	0.0152 (3)	
C25	0.37738 (9)	0.56447 (6)	0.54600 (11)	0.0155 (3)	
H25	0.3850	0.5832	0.6270	0.019*	
C26	0.36546 (9)	0.61053 (6)	0.44566 (11)	0.0154 (3)	
C27	0.35322 (10)	0.58073 (6)	0.32652 (12)	0.0182 (3)	
H27	0.3446	0.6111	0.2564	0.022*	
C28	0.35334 (10)	0.50898 (7)	0.30850 (12)	0.0190 (3)	
H28	0.3444	0.4901	0.2271	0.023*	
C28A	0.36677 (9)	0.46452 (6)	0.41131 (11)	0.0159 (3)	
C221	0.37034 (9)	0.27309 (6)	0.43493 (11)	0.0156 (3)	
C222	0.38561 (10)	0.15314 (6)	0.49520 (11)	0.0187 (3)	
H22A	0.4129	0.1431	0.4289	0.022*	
H22B	0.3154	0.1405	0.4615	0.022*	
C223	0.43928 (11)	0.11188 (7)	0.61367 (12)	0.0211 (3)	
H22C	0.4313	0.0613	0.5947	0.032*	
H22D	0.4127	0.1233	0.6791	0.032*	
H22E	0.5089	0.1240	0.6446	0.032*	
C261	0.36521 (9)	0.68856 (6)	0.46004 (11)	0.0146 (3)	
C262	0.31923 (9)	0.73240 (6)	0.35536 (11)	0.0163 (3)	
H262	0.2869	0.7120	0.2738	0.020*	
C263	0.31995 (9)	0.80523 (6)	0.36846 (11)	0.0167 (3)	
H263	0.2885	0.8336	0.2955	0.020*	
C264	0.36574 (9)	0.83778 (6)	0.48628 (11)	0.0165 (3)	
C265	0.41121 (10)	0.79387 (6)	0.59067 (11)	0.0175 (3)	
H265	0.4428	0.8144	0.6723	0.021*	
C266	0.41138 (9)	0.72107 (7)	0.57822 (11)	0.0166 (3)	
H266	0.4434	0.6928	0.6512	0.020*	
C267	0.36524 (11)	0.91687 (6)	0.49995 (12)	0.0211 (3)	
H26A	0.3662	0.9391	0.4221	0.032*	
H26B	0.4231	0.9316	0.5725	0.032*	
H26C	0.3061	0.9313	0.5141	0.032*	

### (1) Ethyl 6-(4-methylphenyl)-4-oxo-4H-chromene-2-carboxylate Atomic displacement parameters (Å^2^)

**Table:**

	*U*^11^	*U*^22^	*U*^33^	*U*^12^	*U*^13^	*U*^23^
O11	0.0239 (5)	0.0110 (4)	0.0160 (4)	0.0018 (4)	0.0083 (4)	0.0015 (3)
O14	0.0381 (6)	0.0187 (5)	0.0180 (4)	−0.0004 (4)	0.0138 (4)	−0.0006 (4)
O121	0.0209 (5)	0.0164 (4)	0.0209 (4)	0.0001 (4)	0.0103 (4)	−0.0004 (3)
O122	0.0215 (5)	0.0103 (4)	0.0174 (4)	0.0006 (3)	0.0079 (4)	0.0009 (3)
C12	0.0135 (6)	0.0129 (6)	0.0182 (6)	−0.0002 (5)	0.0044 (5)	0.0023 (4)
C13	0.0154 (7)	0.0149 (6)	0.0175 (6)	−0.0010 (5)	0.0053 (5)	0.0023 (5)
C14	0.0173 (7)	0.0157 (6)	0.0168 (6)	−0.0006 (5)	0.0063 (5)	−0.0002 (5)
C14A	0.0131 (6)	0.0145 (6)	0.0167 (6)	0.0002 (5)	0.0047 (5)	0.0006 (5)
C15	0.0155 (6)	0.0157 (6)	0.0155 (6)	−0.0011 (5)	0.0058 (5)	−0.0015 (5)
C16	0.0123 (6)	0.0138 (6)	0.0170 (6)	−0.0001 (5)	0.0042 (5)	−0.0010 (4)
C17	0.0210 (7)	0.0138 (6)	0.0163 (6)	0.0017 (5)	0.0065 (5)	0.0023 (4)
C18	0.0228 (7)	0.0158 (6)	0.0149 (6)	0.0022 (5)	0.0076 (5)	−0.0006 (5)
C18A	0.0145 (7)	0.0111 (6)	0.0190 (6)	0.0008 (5)	0.0059 (5)	−0.0011 (4)
C121	0.0123 (6)	0.0139 (6)	0.0175 (6)	0.0001 (5)	0.0033 (5)	0.0005 (5)
C122	0.0224 (7)	0.0106 (6)	0.0186 (6)	0.0017 (5)	0.0071 (5)	−0.0009 (4)
C123	0.0317 (8)	0.0125 (6)	0.0231 (6)	0.0001 (6)	0.0109 (6)	0.0015 (5)
C161	0.0136 (6)	0.0125 (6)	0.0140 (5)	0.0001 (5)	0.0024 (5)	−0.0006 (4)
C162	0.0161 (7)	0.0165 (6)	0.0159 (6)	0.0015 (5)	0.0065 (5)	−0.0012 (5)
C163	0.0180 (7)	0.0146 (6)	0.0172 (6)	−0.0015 (5)	0.0059 (5)	0.0008 (5)
C164	0.0152 (7)	0.0136 (6)	0.0155 (6)	0.0007 (5)	0.0005 (5)	−0.0020 (4)
C165	0.0155 (7)	0.0177 (6)	0.0151 (6)	0.0014 (5)	0.0047 (5)	−0.0032 (5)
C166	0.0155 (7)	0.0164 (6)	0.0140 (5)	−0.0009 (5)	0.0042 (5)	−0.0003 (4)
C167	0.0241 (8)	0.0141 (6)	0.0237 (6)	0.0007 (5)	0.0084 (6)	−0.0017 (5)
O21	0.0246 (5)	0.0114 (4)	0.0164 (4)	0.0006 (4)	0.0082 (4)	0.0010 (3)
O24	0.0415 (7)	0.0190 (5)	0.0152 (4)	−0.0008 (4)	0.0091 (4)	−0.0007 (3)
O221	0.0232 (5)	0.0172 (5)	0.0163 (4)	0.0007 (4)	0.0046 (4)	−0.0006 (3)
O222	0.0224 (5)	0.0109 (4)	0.0163 (4)	0.0013 (4)	0.0053 (4)	0.0008 (3)
C22	0.0137 (6)	0.0134 (6)	0.0167 (6)	0.0003 (5)	0.0040 (5)	0.0025 (4)
C23	0.0160 (7)	0.0151 (6)	0.0166 (6)	0.0000 (5)	0.0037 (5)	0.0028 (5)
C24	0.0169 (7)	0.0168 (6)	0.0154 (6)	−0.0008 (5)	0.0036 (5)	0.0012 (5)
C24A	0.0130 (6)	0.0152 (6)	0.0156 (6)	0.0000 (5)	0.0031 (5)	0.0011 (4)
C25	0.0155 (7)	0.0155 (6)	0.0147 (5)	−0.0011 (5)	0.0047 (5)	−0.0018 (4)
C26	0.0126 (6)	0.0162 (6)	0.0174 (6)	−0.0004 (5)	0.0055 (5)	−0.0008 (5)
C27	0.0226 (7)	0.0149 (6)	0.0188 (6)	0.0012 (5)	0.0096 (5)	0.0028 (5)
C28	0.0257 (7)	0.0165 (6)	0.0161 (6)	0.0003 (5)	0.0092 (5)	−0.0010 (5)
C28A	0.0154 (7)	0.0130 (6)	0.0196 (6)	0.0008 (5)	0.0068 (5)	−0.0006 (5)
C221	0.0130 (6)	0.0151 (6)	0.0186 (6)	0.0004 (5)	0.0055 (5)	0.0014 (5)
C222	0.0235 (7)	0.0114 (6)	0.0202 (6)	−0.0015 (5)	0.0070 (5)	−0.0021 (5)
C223	0.0280 (8)	0.0142 (6)	0.0222 (6)	0.0005 (5)	0.0107 (6)	0.0024 (5)
C261	0.0139 (6)	0.0138 (6)	0.0179 (6)	0.0006 (5)	0.0082 (5)	−0.0001 (4)
C262	0.0149 (7)	0.0181 (6)	0.0157 (6)	−0.0011 (5)	0.0056 (5)	−0.0021 (5)
C263	0.0158 (7)	0.0170 (6)	0.0168 (6)	0.0019 (5)	0.0056 (5)	0.0029 (5)
C264	0.0151 (7)	0.0158 (6)	0.0207 (6)	−0.0004 (5)	0.0090 (5)	−0.0013 (5)
C265	0.0174 (7)	0.0175 (6)	0.0171 (6)	−0.0009 (5)	0.0057 (5)	−0.0039 (5)
C266	0.0157 (7)	0.0179 (6)	0.0159 (6)	0.0014 (5)	0.0054 (5)	0.0020 (5)
C267	0.0253 (8)	0.0148 (6)	0.0228 (6)	0.0002 (5)	0.0085 (6)	−0.0023 (5)

### (1) Ethyl 6-(4-methylphenyl)-4-oxo-4H-chromene-2-carboxylate Geometric parameters (Å, º)

**Table:**

O11—C12	1.3551 (14)	O21—C22	1.3561 (14)
O11—C18A	1.3769 (14)	O21—C28A	1.3770 (14)
O14—C14	1.2299 (14)	O24—C24	1.2282 (14)
O121—C121	1.2057 (14)	O221—C221	1.2025 (14)
O122—C121	1.3258 (14)	O222—C221	1.3274 (14)
O122—C122	1.4597 (14)	O222—C222	1.4625 (14)
C12—C13	1.3418 (16)	C22—C23	1.3403 (16)
C12—C121	1.5046 (17)	C22—C221	1.5056 (17)
C13—C14	1.4606 (17)	C23—C24	1.4615 (17)
C13—H13	0.9500	C23—H23	0.9500
C14—C14A	1.4785 (16)	C24—C24A	1.4777 (16)
C14A—C18A	1.3936 (16)	C24A—C28A	1.3911 (16)
C14A—C15	1.4061 (16)	C24A—C25	1.4049 (17)
C15—C16	1.3869 (16)	C25—C26	1.3904 (17)
C15—H15	0.9500	C25—H25	0.9500
C16—C17	1.4126 (16)	C26—C27	1.4095 (16)
C16—C161	1.4875 (16)	C26—C261	1.4885 (16)
C17—C18	1.3729 (17)	C27—C28	1.3757 (17)
C17—H17	0.9500	C27—H27	0.9500
C18—C18A	1.3877 (16)	C28—C28A	1.3895 (17)
C18—H18	0.9500	C28—H28	0.9500
C122—C123	1.5001 (17)	C222—C223	1.4998 (17)
C122—H12A	0.9900	C222—H22A	0.9900
C122—H12B	0.9900	C222—H22B	0.9900
C123—H12C	0.9800	C223—H22C	0.9800
C123—H12D	0.9800	C223—H22D	0.9800
C123—H12E	0.9800	C223—H22E	0.9800
C161—C166	1.3973 (17)	C261—C266	1.3989 (16)
C161—C162	1.3979 (17)	C261—C262	1.3995 (16)
C162—C163	1.3893 (17)	C262—C263	1.3885 (17)
C162—H162	0.9500	C262—H262	0.9500
C163—C164	1.3924 (17)	C263—C264	1.3952 (17)
C163—H163	0.9500	C263—H263	0.9500
C164—C165	1.3930 (17)	C264—C265	1.3968 (17)
C164—C167	1.5083 (16)	C264—C267	1.5079 (17)
C165—C166	1.3901 (17)	C265—C266	1.3875 (17)
C165—H165	0.9500	C265—H265	0.9500
C166—H166	0.9500	C266—H266	0.9500
C167—H16A	0.9800	C267—H26A	0.9800
C167—H16B	0.9800	C267—H26B	0.9800
C167—H16C	0.9800	C267—H26C	0.9800
			
C12—O11—C18A	118.08 (9)	C22—O21—C28A	118.25 (9)
C121—O122—C122	114.80 (9)	C221—O222—C222	115.53 (9)
C13—C12—O11	124.30 (11)	C23—C22—O21	124.14 (11)
C13—C12—C121	125.68 (11)	C23—C22—C221	126.14 (11)
O11—C12—C121	109.95 (10)	O21—C22—C221	109.67 (9)
C12—C13—C14	121.37 (11)	C22—C23—C24	121.38 (11)
C12—C13—H13	119.3	C22—C23—H23	119.3
C14—C13—H13	119.3	C24—C23—H23	119.3
O14—C14—C13	123.11 (11)	O24—C24—C23	123.13 (11)
O14—C14—C14A	122.88 (11)	O24—C24—C24A	122.83 (11)
C13—C14—C14A	114.01 (10)	C23—C24—C24A	114.04 (10)
C18A—C14A—C15	118.18 (11)	C28A—C24A—C25	118.24 (11)
C18A—C14A—C14	119.48 (11)	C28A—C24A—C24	119.56 (11)
C15—C14A—C14	122.34 (10)	C25—C24A—C24	122.19 (11)
C16—C15—C14A	121.49 (11)	C26—C25—C24A	121.82 (11)
C16—C15—H15	119.3	C26—C25—H25	119.1
C14A—C15—H15	119.3	C24A—C25—H25	119.1
C15—C16—C17	117.90 (11)	C25—C26—C27	117.43 (11)
C15—C16—C161	122.99 (10)	C25—C26—C261	122.68 (11)
C17—C16—C161	119.11 (10)	C27—C26—C261	119.89 (10)
C18—C17—C16	121.85 (11)	C28—C27—C26	122.09 (11)
C18—C17—H17	119.1	C28—C27—H27	119.0
C16—C17—H17	119.1	C26—C27—H27	119.0
C17—C18—C18A	118.90 (11)	C27—C28—C28A	118.93 (11)
C17—C18—H18	120.6	C27—C28—H28	120.5
C18A—C18—H18	120.6	C28A—C28—H28	120.5
O11—C18A—C18	115.60 (10)	O21—C28A—C28	115.91 (10)
O11—C18A—C14A	122.73 (10)	O21—C28A—C24A	122.62 (10)
C18—C18A—C14A	121.67 (11)	C28—C28A—C24A	121.47 (11)
O121—C121—O122	126.18 (11)	O221—C221—O222	126.22 (11)
O121—C121—C12	123.04 (11)	O221—C221—C22	123.05 (11)
O122—C121—C12	110.77 (10)	O222—C221—C22	110.73 (10)
O122—C122—C123	106.78 (10)	O222—C222—C223	106.56 (10)
O122—C122—H12A	110.4	O222—C222—H22A	110.4
C123—C122—H12A	110.4	C223—C222—H22A	110.4
O122—C122—H12B	110.4	O222—C222—H22B	110.4
C123—C122—H12B	110.4	C223—C222—H22B	110.4
H12A—C122—H12B	108.6	H22A—C222—H22B	108.6
C122—C123—H12C	109.5	C222—C223—H22C	109.5
C122—C123—H12D	109.5	C222—C223—H22D	109.5
H12C—C123—H12D	109.5	H22C—C223—H22D	109.5
C122—C123—H12E	109.5	C222—C223—H22E	109.5
H12C—C123—H12E	109.5	H22C—C223—H22E	109.5
H12D—C123—H12E	109.5	H22D—C223—H22E	109.5
C166—C161—C162	117.85 (11)	C266—C261—C262	117.31 (11)
C166—C161—C16	121.74 (11)	C266—C261—C26	121.49 (11)
C162—C161—C16	120.40 (11)	C262—C261—C26	121.20 (11)
C163—C162—C161	121.06 (11)	C263—C262—C261	121.22 (11)
C163—C162—H162	119.5	C263—C262—H262	119.4
C161—C162—H162	119.5	C261—C262—H262	119.4
C162—C163—C164	121.27 (11)	C262—C263—C264	121.58 (11)
C162—C163—H163	119.4	C262—C263—H263	119.2
C164—C163—H163	119.4	C264—C263—H263	119.2
C163—C164—C165	117.54 (11)	C263—C264—C265	117.07 (11)
C163—C164—C167	120.28 (11)	C263—C264—C267	121.25 (11)
C165—C164—C167	122.16 (11)	C265—C264—C267	121.68 (11)
C166—C165—C164	121.70 (11)	C266—C265—C264	121.70 (11)
C166—C165—H165	119.2	C266—C265—H265	119.1
C164—C165—H165	119.2	C264—C265—H265	119.1
C165—C166—C161	120.59 (11)	C265—C266—C261	121.12 (11)
C165—C166—H166	119.7	C265—C266—H266	119.4
C161—C166—H166	119.7	C261—C266—H266	119.4
C164—C167—H16A	109.5	C264—C267—H26A	109.5
C164—C167—H16B	109.5	C264—C267—H26B	109.5
H16A—C167—H16B	109.5	H26A—C267—H26B	109.5
C164—C167—H16C	109.5	C264—C267—H26C	109.5
H16A—C167—H16C	109.5	H26A—C267—H26C	109.5
H16B—C167—H16C	109.5	H26B—C267—H26C	109.5
			
C18A—O11—C12—C13	−0.81 (19)	C28A—O21—C22—C23	−0.43 (19)
C18A—O11—C12—C121	−177.93 (10)	C28A—O21—C22—C221	177.20 (10)
O11—C12—C13—C14	−0.6 (2)	O21—C22—C23—C24	−0.4 (2)
C121—C12—C13—C14	176.04 (12)	C221—C22—C23—C24	−177.63 (12)
C12—C13—C14—O14	−179.03 (13)	C22—C23—C24—O24	−179.71 (13)
C12—C13—C14—C14A	1.46 (18)	C22—C23—C24—C24A	0.44 (18)
O14—C14—C14A—C18A	179.54 (12)	O24—C24—C24A—C28A	−179.56 (13)
C13—C14—C14A—C18A	−0.96 (17)	C23—C24—C24A—C28A	0.29 (18)
O14—C14—C14A—C15	−0.7 (2)	O24—C24—C24A—C25	−0.7 (2)
C13—C14—C14A—C15	178.85 (12)	C23—C24—C24A—C25	179.18 (12)
C18A—C14A—C15—C16	−0.24 (19)	C28A—C24A—C25—C26	−0.31 (19)
C14—C14A—C15—C16	179.95 (12)	C24—C24A—C25—C26	−179.21 (12)
C14A—C15—C16—C17	0.05 (19)	C24A—C25—C26—C27	0.75 (19)
C14A—C15—C16—C161	179.98 (12)	C24A—C25—C26—C261	−179.29 (12)
C15—C16—C17—C18	0.36 (19)	C25—C26—C27—C28	−0.3 (2)
C161—C16—C17—C18	−179.57 (12)	C261—C26—C27—C28	179.72 (12)
C16—C17—C18—C18A	−0.6 (2)	C26—C27—C28—C28A	−0.5 (2)
C12—O11—C18A—C18	−178.91 (11)	C22—O21—C28A—C28	−178.28 (11)
C12—O11—C18A—C14A	1.31 (18)	C22—O21—C28A—C24A	1.20 (18)
C17—C18—C18A—O11	−179.42 (11)	C27—C28—C28A—O21	−179.49 (12)
C17—C18—C18A—C14A	0.4 (2)	C27—C28—C28A—C24A	1.0 (2)
C15—C14A—C18A—O11	179.80 (11)	C25—C24A—C28A—O21	179.94 (11)
C14—C14A—C18A—O11	−0.39 (19)	C24—C24A—C28A—O21	−1.13 (19)
C15—C14A—C18A—C18	0.03 (19)	C25—C24A—C28A—C28	−0.60 (19)
C14—C14A—C18A—C18	179.84 (12)	C24—C24A—C28A—C28	178.33 (12)
C122—O122—C121—O121	8.49 (18)	C222—O222—C221—O221	−5.78 (19)
C122—O122—C121—C12	−170.74 (10)	C222—O222—C221—C22	174.17 (10)
C13—C12—C121—O121	−156.97 (13)	C23—C22—C221—O221	165.68 (13)
O11—C12—C121—O121	20.10 (17)	O21—C22—C221—O221	−11.89 (17)
C13—C12—C121—O122	22.30 (18)	C23—C22—C221—O222	−14.27 (18)
O11—C12—C121—O122	−160.63 (10)	O21—C22—C221—O222	168.16 (10)
C121—O122—C122—C123	−176.76 (10)	C221—O222—C222—C223	168.94 (11)
C15—C16—C161—C166	−33.27 (18)	C25—C26—C261—C266	24.15 (19)
C17—C16—C161—C166	146.65 (12)	C27—C26—C261—C266	−155.89 (12)
C15—C16—C161—C162	147.55 (12)	C25—C26—C261—C262	−156.33 (12)
C17—C16—C161—C162	−32.53 (18)	C27—C26—C261—C262	23.63 (18)
C166—C161—C162—C163	−0.31 (18)	C266—C261—C262—C263	0.30 (18)
C16—C161—C162—C163	178.90 (11)	C26—C261—C262—C263	−179.24 (11)
C161—C162—C163—C164	0.36 (19)	C261—C262—C263—C264	−0.47 (19)
C162—C163—C164—C165	−0.32 (18)	C262—C263—C264—C265	0.19 (18)
C162—C163—C164—C167	178.21 (12)	C262—C263—C264—C267	−179.32 (12)
C163—C164—C165—C166	0.26 (18)	C263—C264—C265—C266	0.26 (19)
C167—C164—C165—C166	−178.24 (11)	C267—C264—C265—C266	179.76 (12)
C164—C165—C166—C161	−0.23 (19)	C264—C265—C266—C261	−0.4 (2)
C162—C161—C166—C165	0.25 (18)	C262—C261—C266—C265	0.14 (19)
C16—C161—C166—C165	−178.95 (11)	C26—C261—C266—C265	179.68 (12)

### (1) Ethyl 6-(4-methylphenyl)-4-oxo-4H-chromene-2-carboxylate Hydrogen-bond geometry (Å, º)

**Table:**

*D*—H···*A*	*D*—H	H···*A*	*D*···*A*	*D*—H···*A*
C162—H162···*Cg*(C261)	0.95	2.85	3.4914 (15)	126
C262—H262···*Cg*(C161)^i^	0.95	2.84	3.5408 (4)	131

Symmetry code: (i) *x*, −*y*+1/2, *z*−1/2.

### (2) Ethyl 6-(4-fluorophenyl)-4-oxo-4H-chromene-2-carboxylate Crystal data

**Table:**

C_18_H_13_FO_4_	*F*(000) = 648
*M**_r_* = 312.28	*D*_x_ = 1.483 Mg m^−^^3^
Monoclinic, *P*2_1_/*c*	Mo *K*α radiation, λ = 0.71075 Å
*a* = 3.8521 (2) Å	Cell parameters from 15331 reflections
*b* = 20.6970 (15) Å	θ = 2.3–27.5°
*c* = 17.5478 (11) Å	µ = 0.11 mm^−^^1^
β = 91.546 (1)°	*T* = 100 K
*V* = 1398.52 (15) Å^3^	Needle, colourless
*Z* = 4	0.42 × 0.02 × 0.01 mm

### (2) Ethyl 6-(4-fluorophenyl)-4-oxo-4H-chromene-2-carboxylate Data collection

**Table:**

Rigaku Saturn724+ (2x2 bin mode) diffractometer	3177 independent reflections
Radiation source: Sealed Tube	2725 reflections with *I* > 2σ(*I*)
Graphite Monochromator monochromator	*R*_int_ = 0.035
Detector resolution: 28.5714 pixels mm^-1^	θ_max_ = 27.5°, θ_min_ = 2.3°
profile data from ω–scans	*h* = −4→4
Absorption correction: multi-scan *CrystalClear-SM Expert* (Rigaku, 20112)	*k* = −26→26
*T*_min_ = 0.954, *T*_max_ = 0.999	*l* = −22→22
16479 measured reflections	

### (2) Ethyl 6-(4-fluorophenyl)-4-oxo-4H-chromene-2-carboxylate Refinement

**Table:**

Refinement on *F*^2^	0 restraints
Least-squares matrix: full	Hydrogen site location: inferred from neighbouring sites
*R*[*F*^2^ > 2σ(*F*^2^)] = 0.031	H-atom parameters constrained
*wR*(*F*^2^) = 0.087	*w* = 1/[σ^2^(*F*_o_^2^) + (0.0482*P*)^2^ + 0.4787*P*] where *P* = (*F*_o_^2^ + 2*F*_c_^2^)/3
*S* = 0.98	(Δ/σ)_max_ = 0.001
3176 reflections	Δρ_max_ = 0.31 e Å^−^^3^
209 parameters	Δρ_min_ = −0.20 e Å^−^^3^

### (2) Ethyl 6-(4-fluorophenyl)-4-oxo-4H-chromene-2-carboxylate Special details

**Table:**

Geometry. All e.s.d.'s (except the e.s.d. in the dihedral angle between two l.s. planes) are estimated using the full covariance matrix. The cell e.s.d.'s are taken into account individually in the estimation of e.s.d.'s in distances, angles and torsion angles; correlations between e.s.d.'s in cell parameters are only used when they are defined by crystal symmetry. An approximate (isotropic) treatment of cell e.s.d.'s is used for estimating e.s.d.'s involving l.s. planes.

### (2) Ethyl 6-(4-fluorophenyl)-4-oxo-4H-chromene-2-carboxylate Fractional atomic coordinates and isotropic or equivalent isotropic displacement parameters (Å^2^)

**Table:**

	*x*	*y*	*z*	*U*_iso_*/*U*_eq_	
C2	0.3562 (3)	0.34130 (5)	0.52749 (6)	0.0141 (2)	
C3	0.5254 (3)	0.30718 (5)	0.58218 (6)	0.0155 (2)	
H3	0.5714	0.2626	0.5741	0.019*	
C4	0.6397 (3)	0.33746 (5)	0.65381 (6)	0.0149 (2)	
C5	0.6674 (3)	0.44460 (5)	0.72144 (6)	0.0138 (2)	
H5	0.7882	0.4245	0.7630	0.017*	
C4A	0.5615 (3)	0.40718 (5)	0.65824 (6)	0.0135 (2)	
C6	0.5990 (3)	0.51041 (5)	0.72442 (6)	0.0137 (2)	
C7	0.4184 (3)	0.53915 (5)	0.66195 (6)	0.0145 (2)	
H7	0.3699	0.5841	0.6633	0.017*	
C8	0.3108 (3)	0.50362 (5)	0.59912 (6)	0.0147 (2)	
H8	0.1891	0.5237	0.5577	0.018*	
C8A	0.3843 (3)	0.43766 (5)	0.59763 (6)	0.0134 (2)	
C21	0.2302 (3)	0.31555 (5)	0.45161 (6)	0.0143 (2)	
C22	0.2530 (3)	0.22857 (5)	0.36482 (6)	0.0175 (2)	
H22A	0.3781	0.2496	0.3230	0.021*	
H22B	0.0005	0.2343	0.3553	0.021*	
C23	0.3417 (3)	0.15775 (5)	0.36840 (6)	0.0193 (2)	
H23A	0.2795	0.1373	0.3195	0.029*	
H23B	0.2120	0.1372	0.4091	0.029*	
H23C	0.5914	0.1526	0.3789	0.029*	
C61	0.7187 (3)	0.55070 (5)	0.79004 (6)	0.0142 (2)	
C62	0.7275 (3)	0.52672 (5)	0.86460 (6)	0.0169 (2)	
H62	0.6491	0.4840	0.8740	0.020*	
C63	0.8493 (3)	0.56456 (5)	0.92520 (6)	0.0193 (2)	
H63	0.8535	0.5483	0.9759	0.023*	
C64	0.9640 (3)	0.62638 (5)	0.90974 (6)	0.0182 (2)	
C65	0.9542 (3)	0.65260 (5)	0.83766 (6)	0.0179 (2)	
H65	1.0299	0.6956	0.8290	0.021*	
C66	0.8302 (3)	0.61423 (5)	0.77786 (6)	0.0161 (2)	
H66	0.8207	0.6315	0.7277	0.019*	
F64	1.0940 (2)	0.66280 (3)	0.96871 (4)	0.02586 (17)	
O1	0.27388 (19)	0.40483 (3)	0.53315 (4)	0.01485 (17)	
O4	0.7946 (2)	0.30785 (4)	0.70522 (4)	0.02071 (19)	
O21	0.0372 (2)	0.34557 (4)	0.40948 (4)	0.02041 (18)	
O22	0.3576 (2)	0.25727 (4)	0.43820 (4)	0.01680 (17)	

### (2) Ethyl 6-(4-fluorophenyl)-4-oxo-4H-chromene-2-carboxylate Atomic displacement parameters (Å^2^)

**Table:**

	*U*^11^	*U*^22^	*U*^33^	*U*^12^	*U*^13^	*U*^23^
C2	0.0153 (5)	0.0131 (5)	0.0141 (5)	−0.0001 (4)	0.0014 (4)	−0.0013 (4)
C3	0.0180 (5)	0.0132 (5)	0.0151 (5)	0.0016 (4)	−0.0001 (4)	−0.0010 (4)
C4	0.0163 (5)	0.0143 (5)	0.0141 (5)	0.0011 (4)	0.0001 (4)	0.0009 (4)
C5	0.0141 (5)	0.0151 (5)	0.0121 (5)	−0.0001 (4)	−0.0001 (4)	0.0018 (4)
C4A	0.0134 (5)	0.0139 (5)	0.0132 (5)	0.0004 (4)	0.0012 (4)	0.0005 (4)
C6	0.0130 (5)	0.0155 (5)	0.0126 (5)	−0.0014 (4)	0.0011 (4)	−0.0006 (4)
C7	0.0151 (5)	0.0126 (4)	0.0157 (5)	0.0002 (4)	0.0012 (4)	0.0006 (4)
C8	0.0154 (5)	0.0150 (5)	0.0138 (5)	0.0013 (4)	−0.0005 (4)	0.0020 (4)
C8A	0.0140 (5)	0.0149 (5)	0.0113 (4)	−0.0014 (4)	0.0005 (4)	−0.0011 (4)
C21	0.0154 (5)	0.0144 (5)	0.0133 (5)	−0.0012 (4)	0.0014 (4)	0.0006 (4)
C22	0.0198 (5)	0.0194 (5)	0.0130 (5)	0.0002 (4)	−0.0033 (4)	−0.0046 (4)
C23	0.0193 (6)	0.0183 (5)	0.0203 (5)	0.0004 (4)	−0.0002 (4)	−0.0050 (4)
C61	0.0135 (5)	0.0152 (5)	0.0139 (5)	0.0013 (4)	0.0001 (4)	−0.0015 (4)
C62	0.0201 (5)	0.0143 (5)	0.0161 (5)	−0.0008 (4)	0.0006 (4)	0.0004 (4)
C63	0.0250 (6)	0.0199 (5)	0.0130 (5)	0.0004 (4)	−0.0004 (4)	0.0007 (4)
C64	0.0197 (5)	0.0198 (5)	0.0149 (5)	−0.0013 (4)	−0.0010 (4)	−0.0065 (4)
C65	0.0199 (5)	0.0149 (5)	0.0190 (5)	−0.0019 (4)	0.0020 (4)	−0.0017 (4)
C66	0.0180 (5)	0.0159 (5)	0.0144 (5)	0.0004 (4)	0.0009 (4)	0.0005 (4)
F64	0.0363 (4)	0.0244 (3)	0.0167 (3)	−0.0080 (3)	−0.0025 (3)	−0.0070 (3)
O1	0.0197 (4)	0.0123 (3)	0.0123 (3)	0.0017 (3)	−0.0030 (3)	−0.0008 (3)
O4	0.0290 (5)	0.0163 (4)	0.0165 (4)	0.0055 (3)	−0.0062 (3)	0.0002 (3)
O21	0.0265 (4)	0.0183 (4)	0.0161 (4)	0.0045 (3)	−0.0046 (3)	0.0003 (3)
O22	0.0204 (4)	0.0154 (4)	0.0143 (4)	0.0026 (3)	−0.0039 (3)	−0.0039 (3)

### (2) Ethyl 6-(4-fluorophenyl)-4-oxo-4H-chromene-2-carboxylate Geometric parameters (Å, º)

**Table:**

C2—C3	1.3457 (14)	C21—O22	1.3257 (12)
C2—O1	1.3568 (12)	C22—O22	1.4647 (12)
C2—C21	1.5025 (14)	C22—C23	1.5059 (15)
C3—C4	1.4617 (14)	C22—H22A	0.9900
C3—H3	0.9500	C22—H22B	0.9900
C4—O4	1.2315 (13)	C23—H23A	0.9800
C4—C4A	1.4766 (14)	C23—H23B	0.9800
C5—C6	1.3886 (14)	C23—H23C	0.9800
C5—C4A	1.4042 (14)	C61—C62	1.3989 (14)
C5—H5	0.9500	C61—C66	1.4015 (14)
C4A—C8A	1.3984 (14)	C62—C63	1.3920 (15)
C6—C7	1.4135 (14)	C62—H62	0.9500
C6—C61	1.4851 (14)	C63—C64	1.3829 (16)
C7—C8	1.3797 (14)	C63—H63	0.9500
C7—H7	0.9500	C64—F64	1.3646 (12)
C8—C8A	1.3948 (14)	C64—C65	1.3759 (15)
C8—H8	0.9500	C65—C66	1.3903 (14)
C8A—O1	1.3774 (12)	C65—H65	0.9500
C21—O21	1.2065 (13)	C66—H66	0.9500
			
C3—C2—O1	124.47 (9)	O22—C22—H22A	110.2
C3—C2—C21	125.77 (9)	C23—C22—H22A	110.2
O1—C2—C21	109.76 (8)	O22—C22—H22B	110.2
C2—C3—C4	121.14 (9)	C23—C22—H22B	110.2
C2—C3—H3	119.4	H22A—C22—H22B	108.5
C4—C3—H3	119.4	C22—C23—H23A	109.5
O4—C4—C3	123.05 (9)	C22—C23—H23B	109.5
O4—C4—C4A	122.91 (9)	H23A—C23—H23B	109.5
C3—C4—C4A	114.03 (9)	C22—C23—H23C	109.5
C6—C5—C4A	121.29 (9)	H23A—C23—H23C	109.5
C6—C5—H5	119.4	H23B—C23—H23C	109.5
C4A—C5—H5	119.4	C62—C61—C66	118.41 (9)
C8A—C4A—C5	118.51 (9)	C62—C61—C6	121.68 (9)
C8A—C4A—C4	119.79 (9)	C66—C61—C6	119.91 (9)
C5—C4A—C4	121.69 (9)	C63—C62—C61	120.93 (10)
C5—C6—C7	118.26 (9)	C63—C62—H62	119.5
C5—C6—C61	121.65 (9)	C61—C62—H62	119.5
C7—C6—C61	120.07 (9)	C64—C63—C62	118.27 (10)
C8—C7—C6	121.77 (9)	C64—C63—H63	120.9
C8—C7—H7	119.1	C62—C63—H63	120.9
C6—C7—H7	119.1	F64—C64—C65	118.67 (10)
C7—C8—C8A	118.68 (9)	F64—C64—C63	118.35 (10)
C7—C8—H8	120.7	C65—C64—C63	122.98 (10)
C8A—C8—H8	120.7	C64—C65—C66	117.97 (10)
O1—C8A—C8	116.10 (9)	C64—C65—H65	121.0
O1—C8A—C4A	122.40 (9)	C66—C65—H65	121.0
C8—C8A—C4A	121.50 (9)	C65—C66—C61	121.41 (10)
O21—C21—O22	125.79 (10)	C65—C66—H66	119.3
O21—C21—C2	122.64 (9)	C61—C66—H66	119.3
O22—C21—C2	111.57 (9)	C2—O1—C8A	118.08 (8)
O22—C22—C23	107.55 (8)	C21—O22—C22	115.51 (8)
			
O1—C2—C3—C4	−0.66 (17)	C3—C2—C21—O22	−11.10 (15)
C21—C2—C3—C4	179.33 (10)	O1—C2—C21—O22	168.89 (8)
C2—C3—C4—O4	179.88 (11)	C5—C6—C61—C62	−35.73 (15)
C2—C3—C4—C4A	−1.71 (15)	C7—C6—C61—C62	145.98 (11)
C6—C5—C4A—C8A	0.07 (15)	C5—C6—C61—C66	143.58 (11)
C6—C5—C4A—C4	178.64 (10)	C7—C6—C61—C66	−34.71 (15)
O4—C4—C4A—C8A	−179.98 (10)	C66—C61—C62—C63	−1.07 (16)
C3—C4—C4A—C8A	1.60 (14)	C6—C61—C62—C63	178.25 (10)
O4—C4—C4A—C5	1.48 (16)	C61—C62—C63—C64	−0.46 (17)
C3—C4—C4A—C5	−176.94 (9)	C62—C63—C64—F64	−177.91 (10)
C4A—C5—C6—C7	0.08 (15)	C62—C63—C64—C65	1.81 (18)
C4A—C5—C6—C61	−178.24 (9)	F64—C64—C65—C66	178.21 (10)
C5—C6—C7—C8	−0.02 (16)	C63—C64—C65—C66	−1.50 (17)
C61—C6—C7—C8	178.33 (10)	C64—C65—C66—C61	−0.15 (16)
C6—C7—C8—C8A	−0.20 (16)	C62—C61—C66—C65	1.39 (16)
C7—C8—C8A—O1	−179.34 (9)	C6—C61—C66—C65	−177.94 (10)
C7—C8—C8A—C4A	0.36 (16)	C3—C2—O1—C8A	3.16 (15)
C5—C4A—C8A—O1	179.38 (9)	C21—C2—O1—C8A	−176.84 (8)
C4—C4A—C8A—O1	0.79 (15)	C8—C8A—O1—C2	176.53 (9)
C5—C4A—C8A—C8	−0.30 (15)	C4A—C8A—O1—C2	−3.17 (14)
C4—C4A—C8A—C8	−178.89 (10)	O21—C21—O22—C22	0.82 (15)
C3—C2—C21—O21	168.95 (11)	C2—C21—O22—C22	−179.12 (8)
O1—C2—C21—O21	−11.05 (14)	C23—C22—O22—C21	−165.47 (9)

### (2) Ethyl 6-(4-fluorophenyl)-4-oxo-4H-chromene-2-carboxylate Hydrogen-bond geometry (Å, º)

**Table:**

*D*—H···*A*	*D*—H	H···*A*	*D*···*A*	*D*—H···*A*
C7—H7···O21^i^	0.95	2.47	3.1977 (13)	133
C65—H65···O4^ii^	0.95	2.50	3.4447 (13)	175
C66—H66···O21^iii^	0.95	2.53	3.4425 (13)	162

Symmetry codes: (i) −*x*, −*y*+1, −*z*+1; (ii) −*x*+2, *y*+1/2, −*z*+3/2; (iii) −*x*+1, −*y*+1, −*z*+1.
