# Supplementary material for: Crystal structures of ethyl 6-(4-methyl­phen­yl)-4-oxo-4H-chromene-2-carboxyl­ate and ethyl 6-(4-fluoro­phen­yl)-4-oxo-4H-chromene-2-carboxyl­ate
Source: Acta Crystallogr E Crystallogr Commun. 2016 Jan 1;72(Pt 1):8–13. doi: 10.1107/S2056989015022781 (PMC4704759; doi:10.1107/S2056989015022781)
Supplement: Supplementary file 6 [file e-72-00008-sup6.pdf]

Supplementary Data for HB7543

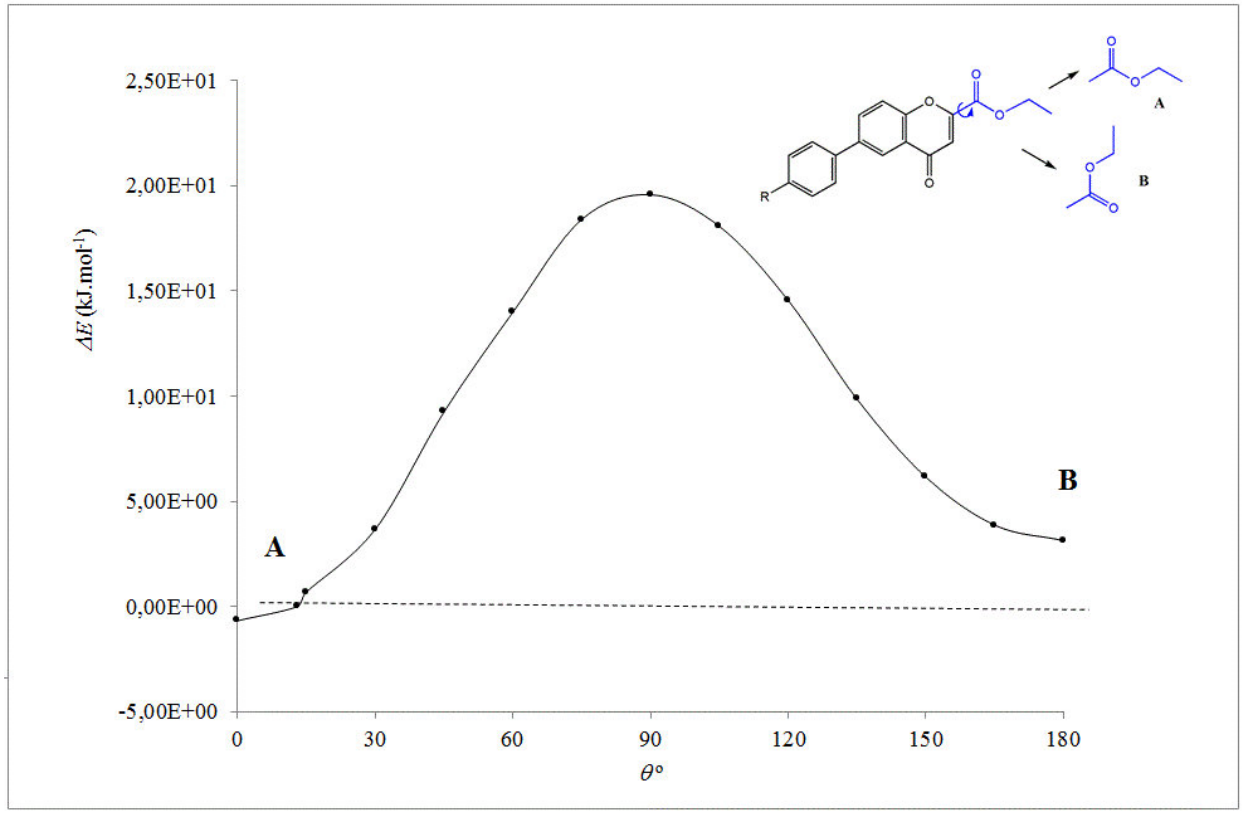

Figure 10 (supplementary): calculated gas-phase energies for the possible conformations for the studied compounds related to a hemisphere rotation of the ester group with respect to the chromone residue. Those lie between the boundary conformation A where the carbonyl groups are *trans*-related and conformation B where they are *cis*-related.

Gas-phase *ab initio* single point calculations for compound (**2**) for geometries between conformations A and B were carried out at the B3LYP/6-311+G(d) level of theory and are referred to the single point energy of compound (**2**) with the geometry obtained by X-ray analysis, $\theta = 13°$. The B3LYP model combines the hybrid exchange functional of Becke (1997) with the gradient-correlation functional of Lee *et al*. (1988) and the split-valence polarized 6-311+G(d, p) basis set (Hehre *et al*., 1988), level of theory as implemented in Gaussian 03 (Frisch *et al*., 2004).

References

Becke, A. D. (1997). *J. Chem. Phys*. **107**, 8554–8560.

Frisch, M. J., Trucks, G. W., Schlegel, H. B., Scuseria, G. E., Robb, M. A., Cheeseman, J. R., Montgomery, J. A. Jr, Vreven, T., Kudin, K. N., Burant, J. C., Millam, J. M., Iyengar, S. S., Tomasi, J., Barone, V., Mennucci, B., Cossi, M., Scalmani, G., Rega, N., Petersson, G. A., Nakatsuji, H., Hada, M., Ehara, M., Toyota, K., Fukuda, R., Hasegawa, J., Ishida, M., Nakajima, T., Honda, Y., Kitao, O., Nakai, H., Klene, M., Li, X., Knox, J. E., Hratchian, H. P., Cross, J. B., Bakken, V., Adamo, C., Jaramillo, J., Gomperts, R., Stratmann, R. E., Yazyev, O., Austin, A. J., Cammi, R., Pomelli, C., Ochterski, J. W., Ayala, P. Y., Morokuma, K., Voth, G. A., Salvador, P., Dannenberg, J. J., Zakrzewski, V. G., Dapprich, S., Daniels, A. D., Strain, M. C., Farkas, O., Malick, D. K., Rabuck, A. D., Raghavachari, K., Foresman, J. B., Ortiz, J. V., Cui, Q., Baboul, A. G., Clifford, J., Cioslowski, S., Stefanov, B. B., Liu, G., Liashenko, A., Piskorz, P., Komaromi, I., Martin, R. L., Fox, D. J., Keith, T., Al-Laham, M. A., Peng, C. Y., Nanayakkara, A., Challacombe, A., Gill, P. M. W., Johnson, B., Chen, W., Wong, M. W., Gonzalez, C. & Pople, J. A. (2004). *GAUSSIAN 03, Revision D. 02*, Gaussian, Inc., Wallingford, Connecticut, USA.

Hehre, W. J., Radom, L., Schleyer, P. V. R. & Pople, J. A. (1986). *Ab Initio Molecular Orbital Theory*, Wiley, New York.

Lee, C., Yang, W. & Parr, G.R. (1988). *Phys. Rev. B*, **37**, 785–789.
